# Prevention of chemotherapy drug-mediated human hair follicle damage: combined use of cooling with antioxidant suppresses oxidative stress and prevents matrix keratinocyte cytotoxicity

**DOI:** 10.3389/fphar.2025.1558593

**Published:** 2025-07-08

**Authors:** Khalidah Ibraheem, Adrian Smith, Andrew Collett, Nikolaos T. Georgopoulos

**Affiliations:** ^1^ School of Applied Sciences, University of Huddersfield, Huddersfield, United Kingdom; ^2^ Department of General Surgery, Calderdale and Huddersfield NHS Foundation Trust, Huddersfield, United Kingdom; ^3^ Biomedical Sciences, University of Bolton, Bolton, United Kingdom; ^4^ Biomolecular Sciences Research Centre, School of Biosciences and Chemistry, Sheffield Hallam University, Sheffield, United Kingdom

**Keywords:** chemotherapy, hair follicle, cytotoxicity, alopecia, reactive oxygen species, cooling

## Abstract

**Background:**

Chemotherapy-induced alopecia (CIA) is a distressing side-effect of cancer treatment. Scalp cooling remains the only available method to mitigate CIA, but its effectiveness varies amongst patients. We previously reported that the cytoprotective effects of cooling are temperature dependent. Here we investigated whether blockade of reactive oxygen species (ROS) by antioxidants can potentiate cooling-mediated cytoprotection against chemotherapy-induced damage in human keratinocytes and hair follicles (HFs).

**Methods:**

*In vitro* monocultures of keratinocytes and *ex vivo* HF organ cultures were treated with doxorubicin or 4-hydroxycyclophosphamide (4HC). Cooling conditions tested ranged between 18°C and 26°C. Keratinocyte viability was assessed via MTS assays. HF viability and function were evaluated by hair shaft elongation measurements, hair cycle staging, immunolabelling for proliferation (% Ki-67+ cells) and apoptosis (% TUNEL+ cells), and melanin intensity via histomorphometry. ROS levels were quantified by flow cytometry and spectrophotometrically. The effects of a panel of antioxidants in combination with cooling were assessed.

**Results:**

Chemotherapy agents reduced keratinocyte viability at 37°C by ∼65%–70%, induced HF dystrophy evident by decreased proliferation (9% Ki-67+ cells), increased apoptosis (23% TUNEL+ cells), and reduced anagen (∼10%) and pigmentation. Optimal cooling at 18°C rescued cell viability, significantly (p < 0.001) restored HF proliferation (42% Ki-67+ cells) and pigmentation, suppressed apoptosis (10% TUNEL+ cells), restored anagen (∼70%) and induced a 2-3-fold increase in hair shaft elongation (p < 0.001), whereas cooling at 26°C was only partially effective. ROS generation increased >3-fold following drug exposure and was attenuated by cooling in a temperature-dependent fashion (p < 0.001). Co-treatment with antioxidant (such as N-acetylcysteine) at 26°C restored cell viability to levels comparable with 18°C, normalizing HF proliferation (41% Ki-67+ cells), suppressing apoptosis (8% TUNEL+ cells) and restoring HF function (e.g., hair shaft elongation, p < 0.001).

**Conclusion:**

Cooling effectively suppresses chemotherapy drug-induced cytotoxicity in human keratinocytes and HFs in a temperature-dependent manner. Combination of cooling with antioxidant functionally compensates for inadequate cytoprotection under sub-optimal cooling conditions, as it prevents HF cell apoptosis and maintains HF viability (proliferation) and functionality (hair growth). This combinatorial approach holds translational promise for improving the efficacy and consistency of scalp cooling in preventing CIA, ultimately improving cancer patient quality-of-life during chemotherapy treatment.

## 1 Introduction

Hair loss represents a highly distressing side-effect of chemotherapy (Balagula et al., 2011), with the fear of alopecia causing severe anxiety in cancer patients (Choi et al., 2014; Paterson et al., 2021) and even refusal of chemotherapy treatment in some cases (van Kleffens and van Leeuwen, 2005). Therefore, understanding the mechanisms of chemotherapy-induced alopecia (CIA) and designing effective prevention strategies are critical for cancer patients (Nangia, 2018). The majority of anti-cancer drugs trigger cytotoxicity in the hair follicles (HFs) leading to hair loss (Koppel and Boh, 2001) and toxicity is often hair cycle phase-specific (Dunnill et al., 2018), with taxanes, alkylating agents and anthracyclines being the main CIA-inducers (Paus et al., 2013; Nangia, 2018).

Chemotherapy agents cause cell death (apoptosis) via several cytotoxicity-inducing signalling pathways. Induction of lethal levels of intracellular reactive oxygen species (ROS) represents a common and important mechanism by which many chemotherapy drugs exert their anticancer effects; this includes anthracyclines (doxorubicin/epirubicin) and alkylating agents (cyclophosphamide) (Nicolson, 2010). Notably, ROS-inducing drugs more frequently cause apoptosis in HFs, suggesting a link between ROS and HF damage (Yang et al., 2018). Interestingly, basal ROS levels in the HF appear to be controlled by the master-regulator of redox homeostasis nuclear factor erythroid 2-related factor 2 (Nrf2), which is prominently expressed in HF matrix keratinocytes, whilst activated Nrf2 protects from ROS-mediated lipid peroxidation and catagen induction (Haslam et al., 2017), thus suggesting a critical role for ROS in HF pathophysiology.

Despite efforts to understand the mechanisms of CIA and the potential promise of candidate pharmacological or biological agents (Dunnill et al., 2018; Purba et al., 2019; Haslam et al., 2021; Samra et al., 2024), scalp cooling remains the only regulatory authority-approved approach for which there is extensive clinical evidence that it can suppress or even prevent CIA (Silva et al., 2020). More recent studies have confirmed the ability of scalp cooling to accelerate hair growth recovery (Kinoshita et al., 2019; Bajpai et al., 2020) as well as demonstrating that scalp cooling abrogates permanent chemotherapy-induced alopecia (Kang et al., 2024). Equally importantly, numerous studies have provided clear and unequivocal evidence that there is no association between use of scalp cooling and risk of scalp metastasis (Lemieux et al., 2009; van de Sande et al., 2010; Lemieux et al., 2011), as extensively reviewed (Rugo et al., 2017; Dunnill et al., 2018). Neither is there any link between scalp cooling and survival in women with breast cancer (Lemieux et al., 2015). Therefore, scalp cooling is not only efficacious (Breed et al., 2011), but also demonstrates acceptable tolerability and a clear safety profile. Yet, the overall efficacy of scalp cooling is currently ∼55%, and although this can rise to ∼80–90% depending on the chemotherapy modality (taxanes in particular) (Dunnill et al., 2018), new approaches to enhance the cytoprotective effects of cooling represent an extremely promising strategy to prevent CIA.

Our previous biological studies focusing on the cytoprotective effects of cooling (hypothermia) using *in vitro* models (Al-Tameemi et al., 2014) have provided evidence that the ability to attenuate or prevent drug-mediated cytotoxicity is not merely due to the induction of vasoconstriction in the scalp, as previously hypothesised (Dunnill et al., 2018). Such *in vitro* models, which included human HF-derived outer root sheath keratinocyte (ORSK) and normal human epidermal keratinocyte (NHEK) cultures, demonstrated that the protective effects of cooling are underpinned by additional mechanisms, such as direct attenuation of cellular drug uptake (Dunnill et al., 2020), whilst emphasising the importance of temperature in the efficacy of cooling to combat cytotoxicity.

Here, using such cell models we demonstrate that oxidative stress drives chemotherapy drug-mediated keratinocyte cytotoxicity and optimal cooling conditions (18°C) can prevent the generation of ROS and loss of viability. We show that under sub-optimal cooling conditions (26°C), ROS induction and cytotoxicity are partially prevented; by contrast, the combination of cooling and treatment with a ROS-inactivating antioxidant prevents cytotoxicity. Importantly, to address any limitations of these 2D-culture models, we utilised *ex vivo* human HF mini-organ cultures as a more physiologically relevant model. We show, for the first time, that cooling protects HFs from drug-mediated regression in a temperature-dependent fashion, whilst combination of cooling and ROS-blockade using an antioxidant prevents drug-mediated HF damage. Moreover, we provide extensive evidence that a panel of antioxidants with different mechanisms of action are effective in preventing drug-mediated keratinocyte cytotoxicity in combination with cooling. Our findings not only clearly demonstrate the cytoprotective capacity of cooling, but also provide a novel combinatorial approach with the potential to enhance the clinical efficacy of scalp cooling and minimise the risk of CIA in cancer patients.

## 2 Materials and methods

### 2.1 Culture of human keratinocytes at physiological and cooling conditions

Normal human epidermal keratinocytes (NHEKs) were established using skin specimens from routine surgical procedures with National Health Service Research Ethics Committee approval and informed written consent from patients with no history of skin malignancy. The HaCaT-derivative line “HaCaTa” was established and maintained in KSFM medium supplemented with EGF and BPE (defined as KSFM-complete, KSFMc) (ThermoFisher Scientific, Loughborough, United Kingdom) as previously reported (Al-Tameemi et al., 2014). Primary NHEK cells were isolated and cultured in KSFMc as detailed elsewhere (Dunnill et al., 2020) and used prior to passage 4-5 to maximize proliferative capacity. Cells were routinely maintained at 37°C in a humidified atmosphere of 5% CO_2_. For cooling experiments, a MyTempo™ Mini CO_2_ (Sigma supplied by Merck Lifescience United Kingdom Ltd., Dorset, United Kingdom) incubator permitted cell culture at defined temperature values.

### 2.2 Cell viability assays

The effect of doxorubicin (Doxorubicin hydrochloride, sc-200923, Santa Cruz Biotechnology) and 4-hydroxycyclophosphamide (4HC) (Niomech, Germany) on keratinocyte viability was determined using the CellTiter 96^®^ AQueous One assay (Promega, Southampton, United Kingdom). HaCaTa were seeded at 7,000 cells/well and NHEK at 5,000 cells/well in standard and Cell+ 96-well plates (Sarstedt, Leicester, United Kingdom), respectively, and the cytotoxicity of these chemotherapy drugs was assessed following a 2-h treatment regime, the rationale for which has been extensively described and justified in our previous studies (Al-Tameemi et al., 2014). Treatments with chemotherapy drugs were performed for 2-h at normal (37°C) or cooling conditions (26, 22 or 18°C), in the presence or absence of antioxidants N-Acetyl-L-cysteine (A7250, Sigma), Resveratrol (sc-200808, Santa Cruz), Trolox (238813, Sigma), MitoTEMPO (SML0737, Sigma) and Propyl gallate (48710-100G-F, Sigma). For such experiments, cell cultures were pre-treated for 1-h with antioxidant, prior to the 2-h drug treatment in the presence of the antioxidant. Cells were subsequently rinsed twice with PBS and incubated with fresh medium containing antioxidant for 72-h. CellTiter reagent was then added and after incubation at 37°C for 4-h, absorbance at 492 nm was measured on a FLUOstar OPTIMA (BMG Labtech, Bucks, United Kingdom).

### 2.3 ROS detection

Cells were incubated for 30-min with 1 µM H2DCFDA (ThermoFisher) following a 2-h treatment with doxorubicin or 4HC (at normal or cooling conditions as well as in the presence or absence of antioxidant NAC). For HaCaTa experiments, cells were cultured in 96-well plates and fluorescence detected spectrophotometrically on a FLUOstar OPTIMA as previously (Dunnill et al., 2017; Ibraheem et al., 2022). For NHEK experiments, fluorescence was determined by flow cytometry following acquisition of 10,000 events on a Guava EasyCyte instrument and results analysed using EasyCyte software (Luminex, Amsterdam, Netherlands).

### 2.4 Establishment of human hair follicle cultures and treatments

Human HFs were isolated by microdissection of human temporal scalp skin obtained via facelift surgery (purchased from Caltag Medsystems Ltd., Bucks, United Kingdom) and organ cultures were established based on previously described methods (Langan et al., 2015). Data presented are from a minimum of three donors, each experiment consisted of five technical replicates and each replicate comprised three different cultured HFs (thus n = 15 HFs per donor), unless otherwise stated in the respective figure caption. Upon isolation (Day 0), HFs were cultured in 24-well plates, imaged by phase-contrast microscopy and incubated overnight. On Day 1, culture medium was replaced with fresh medium (Control) or medium containing 30 µM 4HC, 5 mM NAC, or both. HFs were incubated for 2-h at normal (37°C) or cooling conditions (26 or 18°C), before fresh medium was added. Of note, cultures were pre-treated with NAC for 1-h prior to 4HC treatment, following which HFs were rinsed twice with PBS and incubated in fresh medium. On Day 2, cultures were imaged, on Day 3 they were medium-changed, and on Day 4 the HFs were imaged and frozen. HF images were analysed for hair shaft elongation using ImageJ software (https://imagej.net/). HF morphology and staging were also assessed as previously (Bodo et al., 2007).

### 2.5 Preparation of tissue sections, staining and immunolabelling

HF cryosections (7 μm) were prepared as previously described (Langan et al., 2015), before processing for Masson-Fontana histochemistry and Ki-67/TUNEL dual-immunofluorescence microscopy (Haslam et al., 2017; Haslam and Smart, 2019). A Zeiss Axio Imager Z1 was used to image Ki-67/TUNEL sections, images were captured with a Zeiss AxioCam MRm Rev.3 camera and processed using ZEN software (Carl Zeiss Ltd., Herts, United Kingdom). Quantification of Ki-67+ and TUNEL+ cells in areas below Auber’s line was performed using ImageJ according to well-established recommended methodologies (Oh et al., 2016). Widefield microscopy was used for Masson-Fontana imaging, and melanin intensity was quantified for the epithelial region of the HF bulb using ImageJ.

### 2.6 Statistics

Statistical analysis was performed using Minitab v18.1 (Minitab) software. Mean and standard error of the mean (SEM) were used for descriptive purposes and evaluation of significance was calculated by a two-tailed independent Student’s t-test, as deemed suitable by the statistical analysis software following appropriate normality tests (Shapiro-Wilk). For graphical purposes in the captions: *p < 0.05, **p < 0.01 and ***p < 0.001, whilst “NS” denotes non-significance (p > 0.05).

## 3 Results

### 3.1 Induction of ROS by genotoxic chemotherapy agents and its inhibition by cooling (hypothermia) in human keratinocytes

We have previously used *in vitro* models of primary (normal human epidermal keratinocytes, NHEKs) and immortalized adapted-HaCaT cells (“HaCaTa”) (Al-Tameemi et al., 2014) under conditions where they adopt a basal, proliferative phenotype resembling the rapidly-dividing, matrix keratinocytes in human hair follicles (HFs). We demonstrated that a range of cooling conditions (18°C–26°C) effectively block taxane chemotherapy drugs (such as docetaxel) from causing cytotoxicity (Al-Tameemi et al., 2014), including in HF-derived primary follicular matrix keratinocytes. Cooling was also extremely effective at rescuing cells from damage by genotoxic agents such as anthracyclines (doxorubicin) and alkylating agents (cyclophosphamide); yet, it was essential that “optimal” cooling conditions (18°C) were applied for such agents, and in some cases, depending on the drug concentration used, protection was not complete (Al-Tameemi et al., 2014; Dunnill et al., 2020).

**FIGURE 1:**
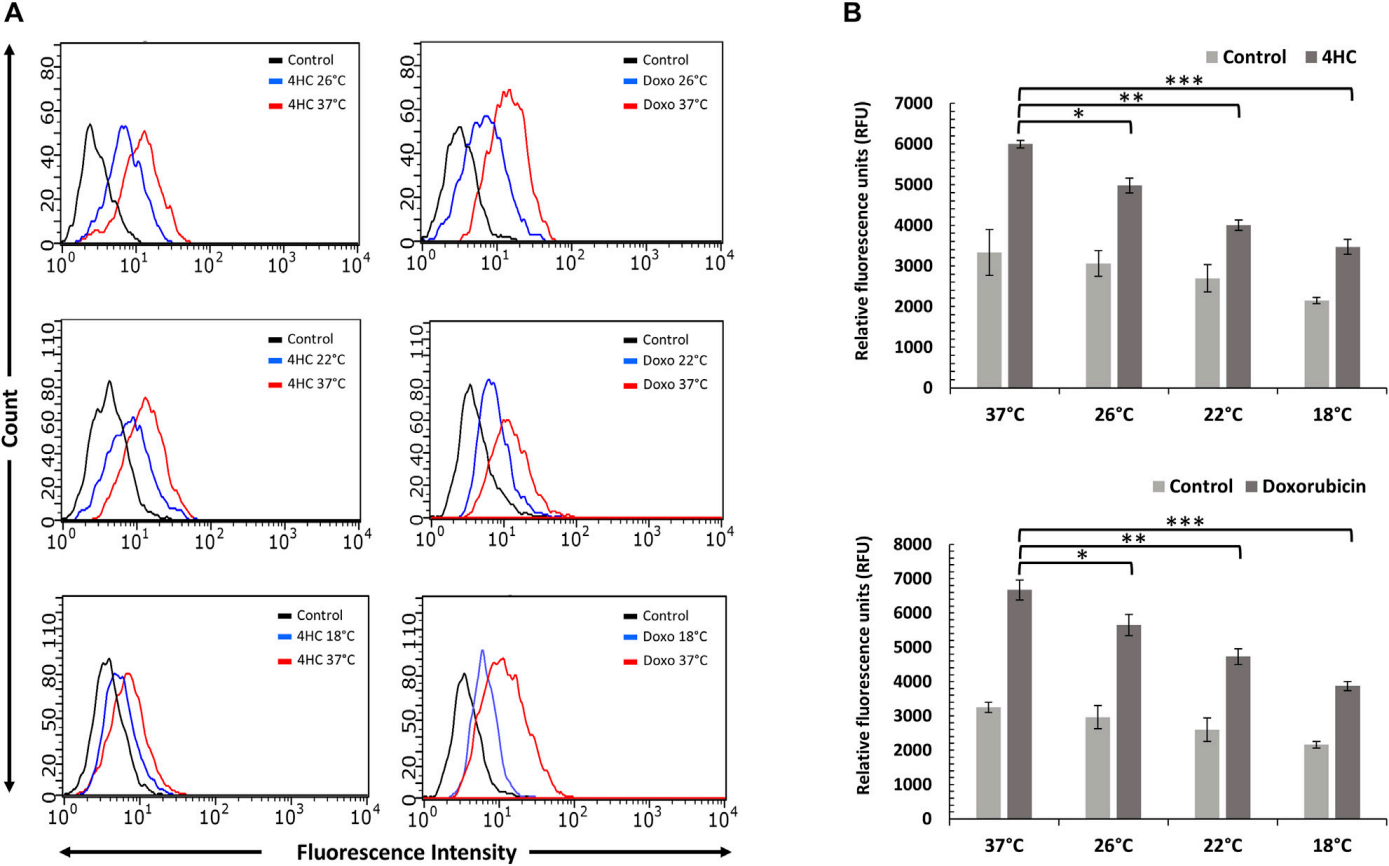
Cooling reduces chemotherapy drug-mediated ROS generation in NHEK and HaCaTa keratinocytes. Cells were treated with 30 µM 4-hydroxycyclophosphamide (4HC) or 1 µM doxorubicin (Doxo) for 2-h alongside untreated cells (Control) at normal (37°C) and at cooling (26°C, 22°C and 18°C) conditions, and ROS generation was detected using H2DCFDA. **(A)** ROS production in NHEK cells was determined by flow cytometry. Overlay histograms of fluorescence intensity are representative of three experiments each consisting of two internal (technical) replicates. For presentation clarity, controls for “37°C only” (not cooling conditions) are shown in the overlay histograms. **(B)** ROS detection in HaCaTa cells was performed spectrophotometrically and expressed as relative fluorescence units (RFUs). Bars show mean RFU (±SEM) for three representative experiments each consisting of four to five technical replicates. Statistical significance (cooling conditions versus 37°C for 4HC treatment) is denoted as *p < 0.05, **p < 0.01 and ***p < 0.001.

A large number of cancer chemotherapy drugs cause cell death, at least partly, by raising intracellular ROS levels, which accounts for their therapeutic (cytotoxic) effect (Yang et al., 2018). We treated NHEKs and HaCaTa keratinocytes with doxorubicin and the active metabolite of cyclophosphamide, 4-hydroxycyclophosphamide (4HC), for 2-h under normal (standard culture) temperature (37°C) and at cooling conditions, and we detected ROS production immediately after treatment. To assess the effect of cooling on ROS production, we tested conditions ranging from optimal temperature (18°C) to cooling conditions exhibiting sub-optimal efficacy (26°C) in protecting from drug-mediated cytotoxicity. As shown in Figure 1, treatment with 4HC and doxorubicin induced rapid ROS production in both NHEK (Figure 1A) and HaCaTa (Figure 1B) cells. However, when chemotherapy drug treatment was carried out under cooling conditions (26, 22°C and 18°C), cooling significantly suppressed ROS generation in a temperature-dependent fashion, more prominently at the optimal (18°C) temperature. Interestingly, cooling alone also curtailed basal ROS production in drug-untreated cultures (Figure 1B). Therefore, cooling suppresses oxidative stress triggered by chemotherapy agents following 2-h of treatment.

### 3.2 Combination of cooling with antioxidant prevents drug-mediated cytotoxicity by suppressing ROS production in human keratinocytes

**FIGURE 2:**
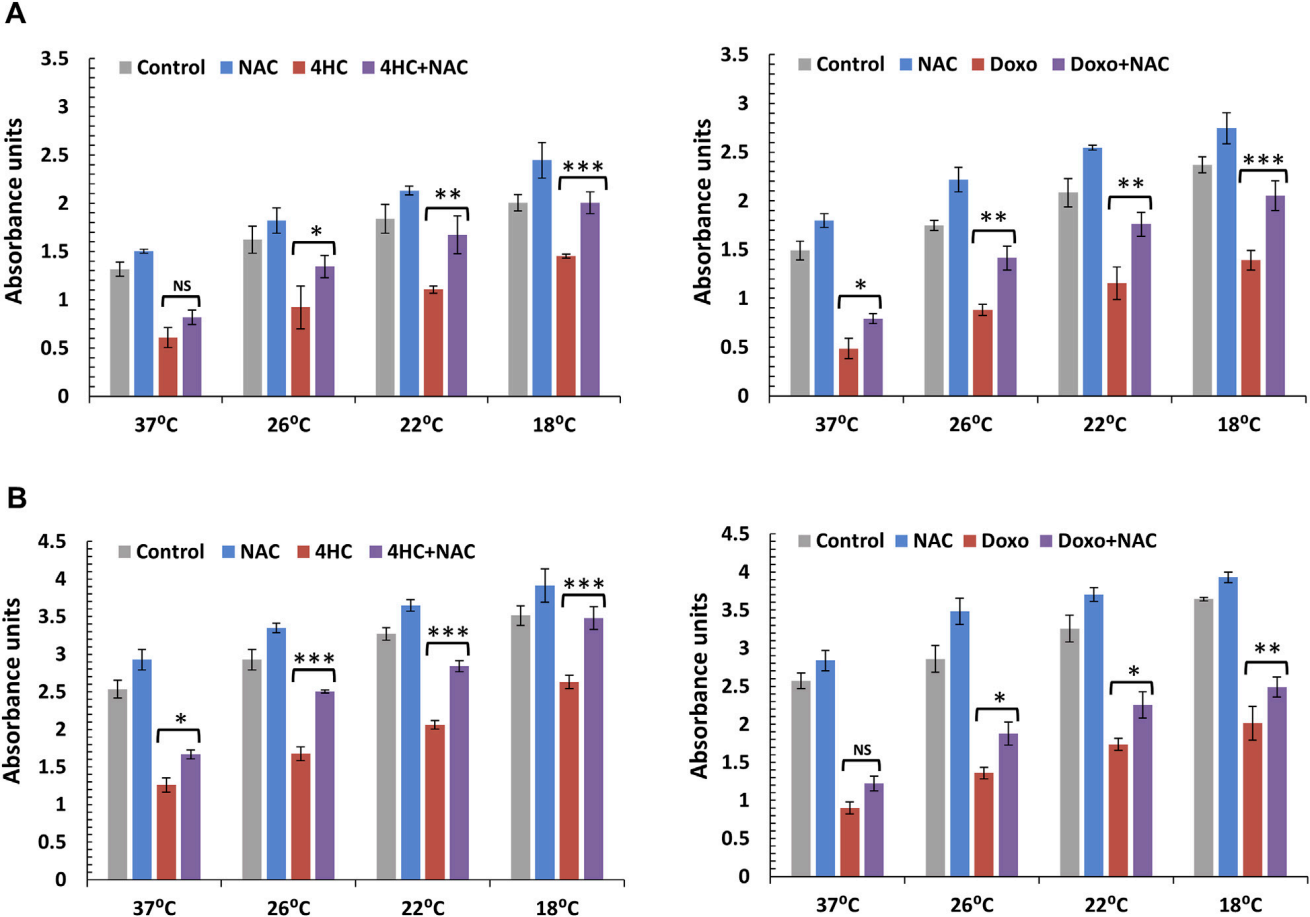
Cytoprotective effect of cooling alone or in combination with the antioxidant NAC against chemotherapy drug-induced cytotoxicity in NHEK and HaCaTa keratinocytes. NHEK **(A)** and HaCaTa **(B)** cells were treated with 30 µM 4-hydroxycyclophosphamide (4HC) (left panels) or 1 µM doxorubicin (Doxo) (right panels) for 2-h in the presence or absence of 400 μM N-Acetyl-L-cysteine (NAC) alongside untreated cells (Control), as well as cultures treated with antioxidant only (as indicated), at normal temperature (37°C) and cooling (26°C, 22°C and 18°C) conditions and cell viability was assessed 72-h post-treatment. Bars show mean absorbance units (±SEM) for three representative experiments each consisting of four to five technical replicates. Statistical significance ([4HC] vs. [4HC + NAC] and [Doxo] vs. [Doxo + NAC]) is denoted as *p < 0.05, **p < 0.01 and ***p < 0.001, whilst NS indicates non-significance (p > 0.05).

Induction of ROS by both chemotherapy agents prompted us to explore the possibility that drug-mediated cytotoxicity could be suppressed in the presence of antioxidant. We used N-Acetyl-L-cysteine (NAC) as a model compound due to its well-characterised, multiple antioxidant properties (Dunnill et al., 2017) and assessed cell viability 72-h post-treatment. Notably, use of NAC alone during chemotherapy drug treatment of NHEK and HaCaTa cells conferred moderate protection from toxicity (Figure 2). By contrast, although cooling alone attenuated drug-mediated cytotoxicity in agreement with our previous findings (Al-Tameemi et al., 2014), combination of cooling and NAC resulted in dramatic protection from cytotoxicity. Strikingly, in primary NHEKs, combination of cooling and NAC (even at sub-optimal temperature) completely prevented drug toxicity, with viability observed being essentially identical to non-drug treated NHEKs (Figure 2A). Our findings were similar (albeit less dramatic for doxorubicin treatment) in HaCaTa cells (Figure 2B).

**FIGURE 3:**
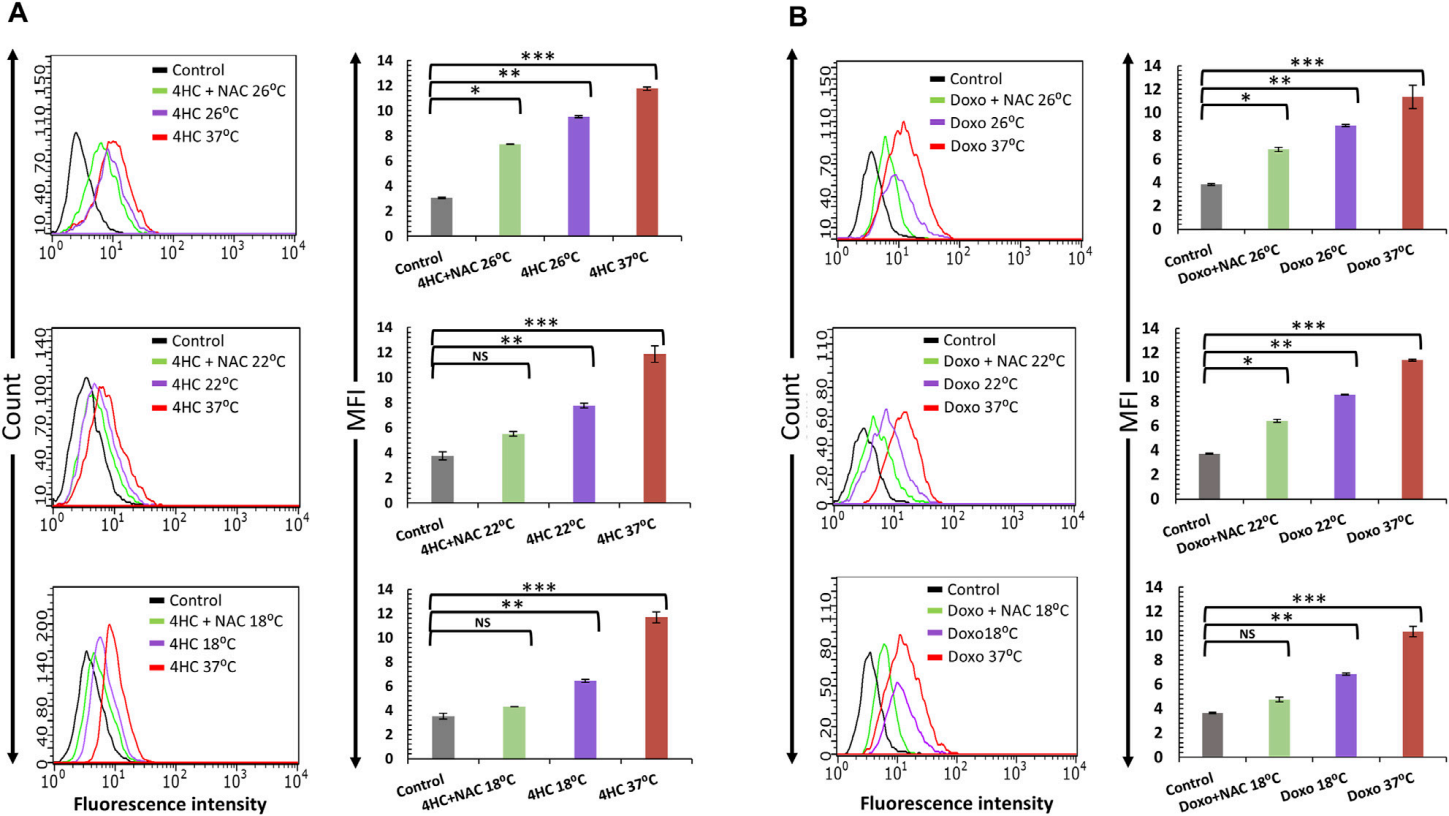
Blockade of chemotherapy drug-induced ROS generation by cooling in combination with antioxidant NAC in NHEK cells. NHEK cells were treated with **(A)** 30 µM 4-hydroxycyclophosphamide (4HC) or **(B)** 1 µM doxorubicin (Doxo) for 2-h in the presence or absence of 400 μM N-Acetyl-L-cysteine (NAC) alongside untreated cells (Control) at normal (37°C) and cooling (26°C, 22°C and 18°C) conditions. ROS production was determined by flow cytometry and data is presented as overlay histograms (left panels), and median fluorescence intensity (MFI) values (±SEM) (right panels), and results are representative of three experiments (donors) each consisting of two technical replicates. Statistical significance is denoted as *p < 0.05, **p < 0.01 and ***p < 0.001, whilst NS indicates non-significance (p > 0.05).

We then sought to determine whether the cytoprotective effect of combination of cooling and antioxidant could be attributed to effects on intracellular ROS generation. Although NAC alone had a modest effect on ROS production by the chemotherapy agents (not shown), combinatorial use of cooling and NAC suppressed ROS production by both 4HC (Figure 3A) and doxorubicin (Figure 3B) in NHEK cells. Notably, our observations of complete blockade of ROS production, particularly at optimal (18°C) cooling conditions, are in concordance with our data on cell viability (Figure 2). Moreover, our findings were similar when such experiments were performed using HaCaTa cells (Supplementary Figure S1). Therefore, the ability of optimal cooling to rescue cells from chemotherapy drug-mediated cytotoxicity and the potentiation of cytoprotection by the antioxidant both coincide with a dramatic reduction in intracellular ROS levels.

### 3.3 Cooling inhibits chemotherapy drug-mediated induction of HF dystrophy

Despite our clinically relevant observations on the cytoprotective properties of cooling using *in vitro* models employing epidermal keratinocytes here and elsewhere (Al-Tameemi et al., 2014) and outer root sheath keratinocyte (ORSK) cultures previously (Dunnill et al., 2020), such models are arguably relatively reductive in nature. Therefore, we utilised human HF cultures as a highly physiologically relevant model to examine, for the first time, the ability of cooling to influence chemotherapy drug-mediated HF damage *ex vivo*. We employed the well-characterised model for CIA involving human HF exposure to 4HC (Bodo et al., 2007; Smart et al., 2019). However, to align with our methodologies for the treatment of cultured keratinocytes, we exposed HFs to 4HC for 2-h. Hair cycle stage (Day 4) and hair shaft elongation (Days 1–4) were then assessed using histomorphometry.

**FIGURE 4:**
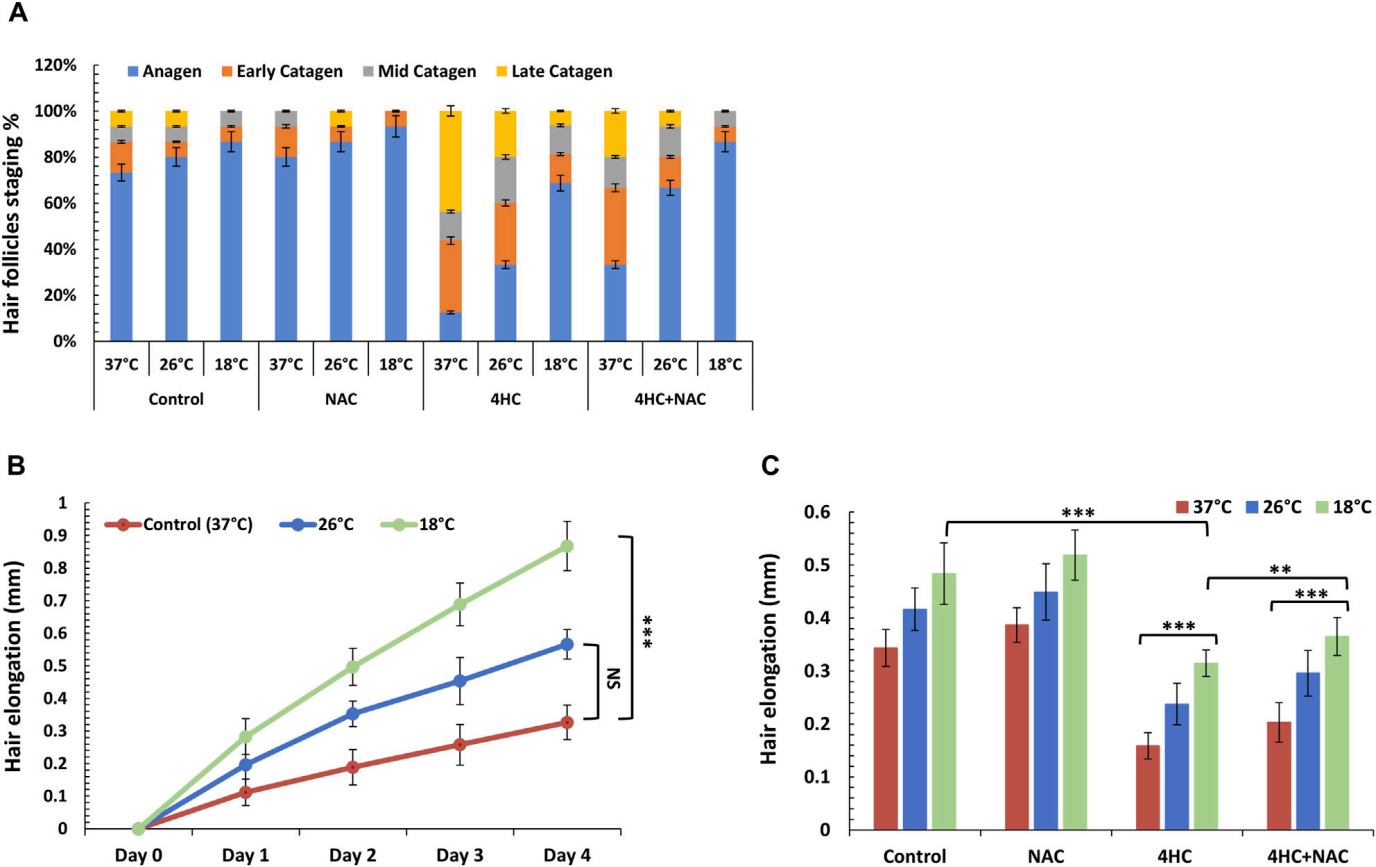
Cooling alone or sub-optimal cooling conditions in combination with antioxidant NAC prevent human HF catagen induction and derepress hair elongation. Human HF organ cultures were treated with 30 µM 4-hydroxycyclophosphamide (4HC) in the presence or absence of 5 mM N-Acetyl-L-cysteine (NAC) alongside untreated cultures (Control) at normal temperature (37°C), as well as at sub-optimal (26°C) and optimal (18°C) cooling conditions (n = 15 HFs, pooled from a minimum of three skin specimens/donors). **(A)** The percentage of HFs at each hair cycle stage was determined on Day 4 post-treatment and results are presented as mean % (±SEM). **(B)** Hair elongation was assessed daily (up to Day 4) for HF cultures treated with 4HC at cooling conditions (26°C and 18°C) vs. control cultures (37°C) and data points represent hair length (mm) (±SEM). **(C)** Hair elongation measurements are presented for Day 4 for HF cultures treated with 4HC in the presence or absence of NAC, at cooling conditions (26°C and 18°C) versus control cultures (37°C), and data expressed as hair length (mm) (±SEM). Statistical significance is denoted as **p < 0.01 and ***p < 0.001, whilst NS indicates non-significance (p > 0.05).

Treatment with 4HC increased the proportion of HFs entering catagen (Figure 4A); however, when treatment was performed under cooling conditions, 4HC-induced HF catagen was reduced (in line with our 72-h cell viability results using cultured keratinocytes), and the prevention of 4HC-induced catagen was temperature-dependent (Figure 4A). Notably, although sub-optimal cooling (26°C) moderately reduced catagen entry, optimal cooling (18°C) markedly attenuated catagen induction (representative examples of images from this analysis are provided in Supplementary Figure S2). These hair cycle staging findings were mirrored by our results from analysis of hair shaft elongation. In particular, whilst 4HC suppressed hair shaft elongation at normal temperature (“Control 37°C”), sub-optimal cooling demonstrated moderate improvement, whereas optimal cooling derepressed hair shaft elongation (Figure 4B). Notably, hair shaft length in drug-treated HFs cooled at 18°C was closely similar to the length observed in drug-untreated HFs at 37°C (Figure 4C). Thus, our observations show, for the first time, that cooling can block chemotherapy drug-mediated HF dystrophy (catagen entry) and permit normal hair shaft elongation.

### 3.4 Combination of cooling with antioxidant prevents HF catagen entry, apoptosis and pigmentary abnormalities induced by chemotherapy

In light of the ability of antioxidant to potentiate the cytoprotective effect of cooling and blockade of drug-mediated keratinocyte toxicity, we investigated the effect of NAC in combination with cooling to modulate HF viability. Strikingly, NAC treatment coupled with optimal cooling fully blocked HF entry into catagen; equally importantly, combination of NAC with sub-optimal (26°C) cooling was equally as effective at preventing catagen entry as optimal (18°C) cooling alone (Figure 4A). Concordantly, co-treatment with NAC also enhanced the ability of cooling to derepress hair elongation (Figure 4C).

**FIGURE 5:**
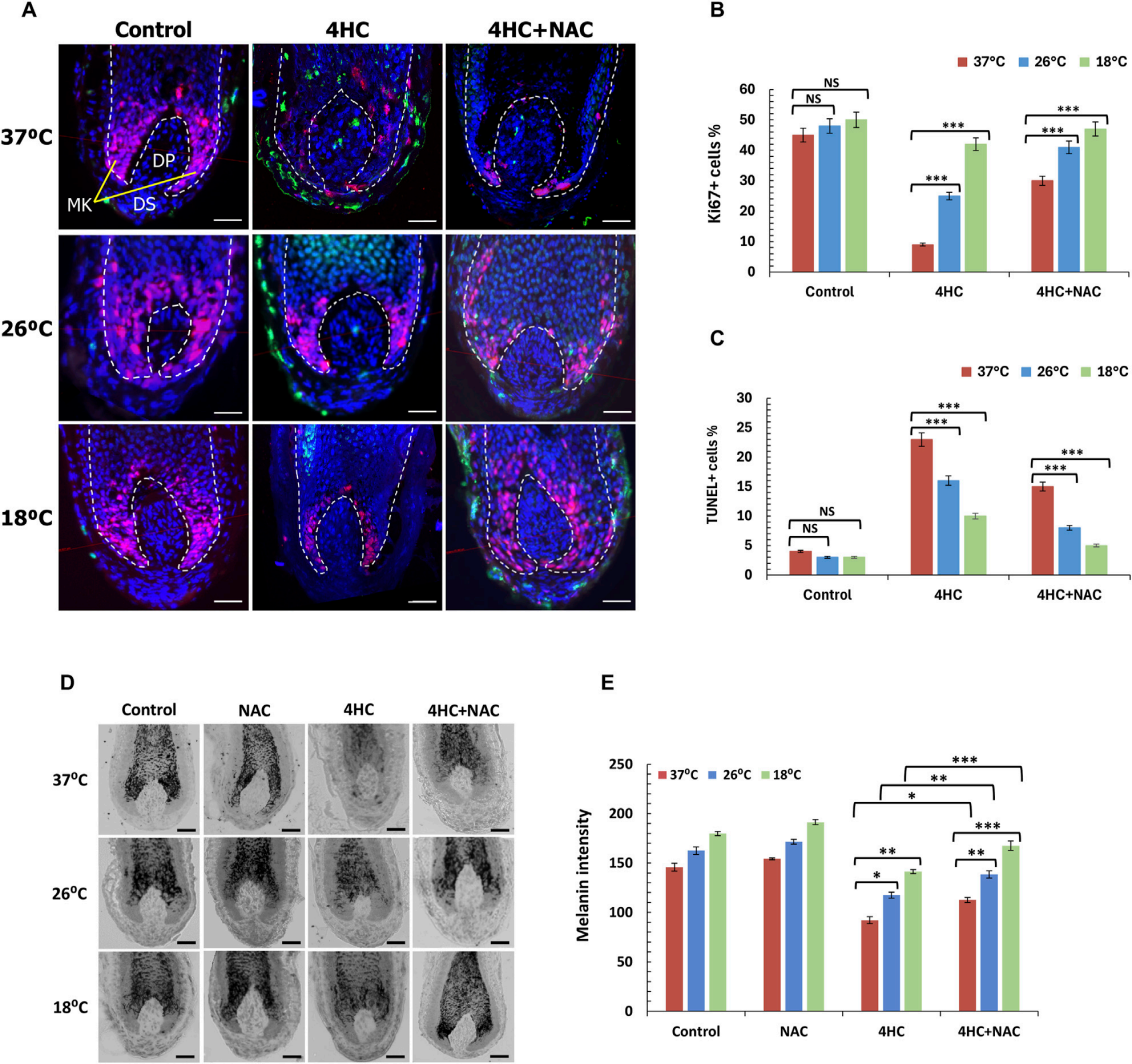
Optimal cooling or sub-optimal cooling in combination with NAC prevent chemotherapy drug-mediated HF growth inhibition and pigmentary abnormalities. HF cultures were treated with 30 µM 4-hydroxycyclophosphamide (4HC) in the presence or absence of 5 mM N-Acetyl-L-cysteine (NAC) alongside untreated cultures (Control) at normal temperature (37°C), as well as at sub-optimal (26°C) and optimal (18°C) cooling conditions followed by a 4-day culture period (n = 15 HFs, pooled from at least three specimens/donors). **(A)** Representative images of Ki-67 and TUNEL labelling of HFs showing Ki-67+ (red) and TUNEL+ (green) cells, with nuclei counter-labelled using DAPI (blue). Scale bars = 50 µm. **(B)** Proliferation of keratinocytes in the HF bulb was assessed by detection of Ki-67+ cells, and bars represent mean % positivity (±SEM). **(C)** Apoptosis was assessed by labelling of TUNEL+ cells and bars represent % positivity (±SEM). Statistical significance ([4HC] vs. [4HC + NAC]) is denoted as ***p < 0.001, whilst NS indicates non-significance (p > 0.05). **(D)** Representative images of Masson-Fontana histochemistry of HFs showing melanin pigmentation and its distribution. Scale bars = 50 µm. **(E)** Melanin intensity was analysed, and bars represent mean intensity (±SEM). Statistical significance (cooling condition vs. 37°C and ([4HC] vs. [4HC + NAC] per temperature) is denoted as *p < 0.05, **p < 0.01 and ***p < 0.001, whilst NS indicates non-significance (p > 0.05).

To reach a deeper understanding of the ability of cooling to protect HFs from chemotherapy drug-induced damage, we performed systematic quantitative immunohistomorphometry analysis to examine proliferation and apoptosis in the HF bulb (Haslam et al., 2017; Smart et al., 2019). 4HC caused a dramatic reduction in cell proliferation, as assessed by the detection of Ki-67+ matrix keratinocytes, and triggered extensive apoptosis evident by the detection of TUNEL+ cells in both the hair bulb (matrix keratinocytes) and the dermal papilla (Figure 5A). Strikingly, cooling suppressed drug-induced apoptosis and restored proliferation, which was particularly dramatic for optimal (18°C) cooling (Figures 5A–C). Inclusion of NAC during cooling significantly potentiated the cytoprotective effect of cooling against drug cytotoxicity (Figures 5A–C), and at optimal cooling conditions full prevention of HF matrix keratinocyte apoptosis was observed.

Furthermore, we tested the ability of cooling to influence abnormalities in melanin distribution and intensity, as such abnormalities are classically indicative of chemotherapy-induced HF dystrophy (Smart et al., 2019). Melanin staining was markedly curtailed following drug treatment, as 4HC treatment resulted in loss of melanin intensity and ectopic melanin granule appearance even above the pigmentary unit (Figures 5D, E). By contrast, cooling rescued from loss of melanin staining and under optimal cooling conditions (18°C) melanin intensity loss was almost completely prevented (Figures 5D, E). Equally importantly, inclusion of NAC during 4HC treatment further enhanced the ability of cooling to prevent the reduction in melanin staining, and combination of cooling with antioxidant appeared to restore both melanin staining intensity and distribution pattern (Figure 5D). Collectively, therefore, our findings demonstrate that cooling significantly suppresses chemotherapy drug-induced HF dystrophy, and combination of cooling with antioxidant prevents loss of HF cell apoptosis and maintains both HF viability (proliferation) and functionality (hair growth).

### 3.5 Assessment of a panel of antioxidants with different mechanisms of action on their ability to enhance the cytoprotective effect of cooling

Our observations using *in vitro* keratinocytes and HF *ex vivo* cultures demonstrated that cooling in conjunction with antioxidant represents a cytoprotective combination that prevents cytotoxicity in HFs. Yet, it was critical to determine whether the observed effect was not specific to the antioxidant NAC and to test the ability of other ROS-scavenging compounds. Thus, we investigated whether other antioxidants could similarly enhance the impact of cooling. Moreover, as NAC was extremely effective against 4HC but evidently less potent against doxorubicin, it was essential to determine whether other ROS inhibitors could have a more significant effect alongside cooling.

**FIGURE 6:**
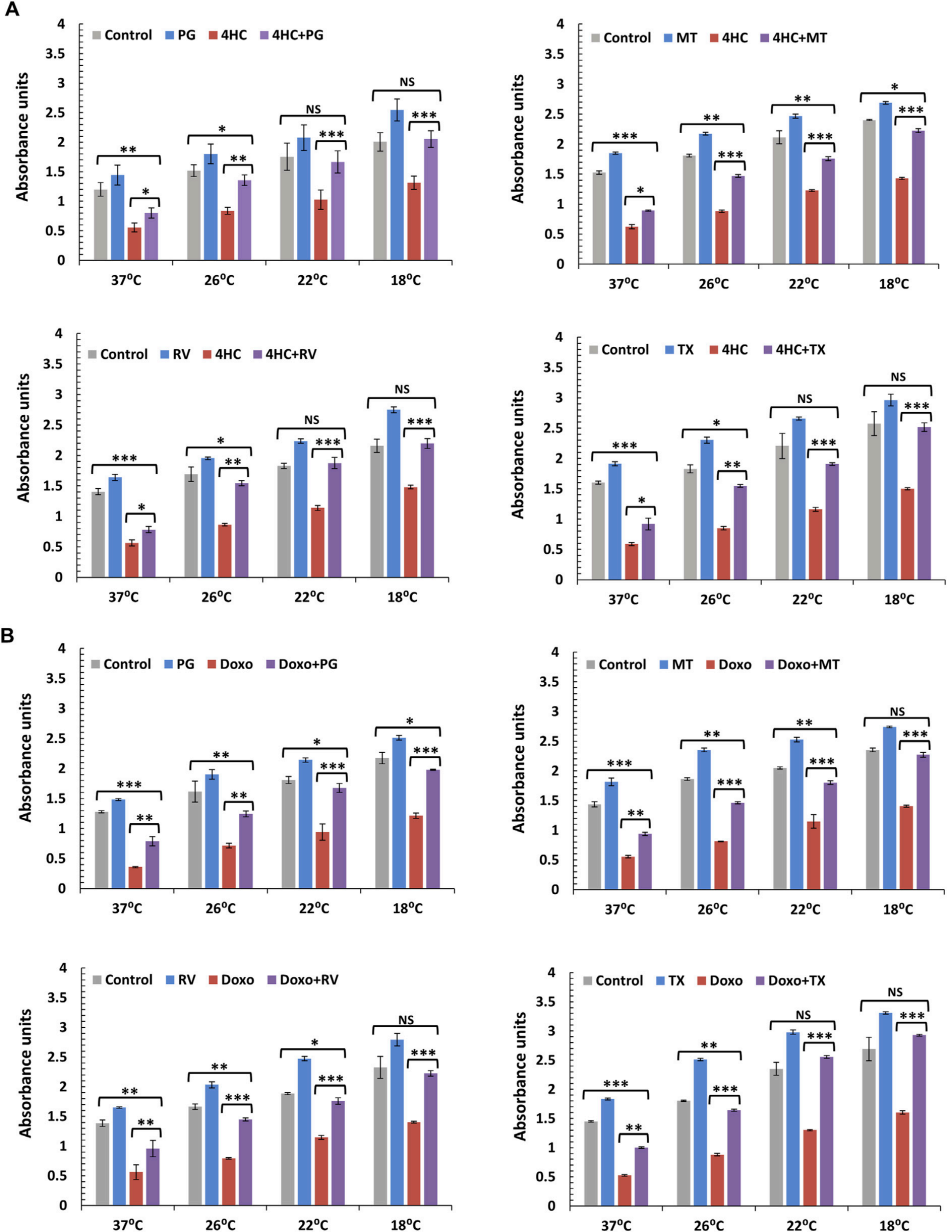
Combination of cooling and a panel of antioxidants with different mechanisms of action protects against chemotherapy drug-induced cytotoxicity in NHEK cells. NHEK cultures were treated with **(A)** 30 µM 4-hydroxycyclophosphamide (4HC) or **(B)** 1 µM doxorubicin (Doxo) for 2-h in the presence or absence of 10 µM Propyl Gallate (PG), 100 µM MitoTEMPO (MT), 10 µM Resveratrol (RV) or 100 µM Trolox (TX) alongside untreated cells (Control) at normal (37°C) and cooling (26°C, 22°C and 18°C) conditions and cell viability was assessed 72-h post-treatment. Bars represent mean absorbance units (±SEM) for three independent experiments (donors) each consisting of four to five technical replicates. Statistical significance ([drug] vs. [drug + antioxidant] and [Control] vs. [drug + antioxidant]) is denoted as *p < 0.05, **p < 0.01 and ***p < 0.001, whilst NS indicates non-significance (p > 0.05).

We systematically investigated a panel of antioxidants (which included natural and synthetic compounds) with different mechanisms of action, and utilised cultured NHEKs as a more versatile model to provide high-throughput testing of such compounds in combination with cooling (optimal antioxidant concentrations used were determined by pre-titration experiments). Unlike antioxidants such as ascorbic acid (vitamin C) and Quercetin that exhibited little efficacy (data not shown), we observed substantial potentiation of the cytoprotective capacity of cooling by the antioxidants Propyl Gallate (PG), MitoTEMPO (MT), Resveratrol (RV) and the water-soluble vitamin E derivative Trolox (TX) against cytotoxicity induced by 4HC (Figure 6A) and doxorubicin (Figure 6B). Although all antioxidants tested showed significant efficacy in combination with cooling, natural compound RV and synthetic molecules MT and TX demonstrated strikingly strong cytoprotective effects (Figure 6B), being equally (if not more) potent than NAC against doxorubicin (Figure 2A). Particularly noteworthy also was the ability of TX to protect against both 4HC and doxorubicin. Finally, our observations in NHEK cells were mirrored by our findings using HaCaTa cells, where use of the antioxidants dramatically compensated for the inability of sub-optimal cooling to cyto-protect (Supplementary Figure S3).

## 4 Discussion

Unravelling the mechanisms of CIA and designing effective prevention strategies are critical, due to the severely traumatic effect of hair loss on cancer patients’ wellbeing as well as the high risk of permanent hair loss (“persistent-CIA,” pCIA) (Chan et al., 2021). Scalp cooling (SC) is currently the only available such method with clinically proven efficacy and clear safety profile. SC protects well against some chemotherapy regimens (mainly taxanes), it accelerates hair regrowth following completion of chemotherapy treatment (Kinoshita et al., 2019; Watanabe et al., 2019; Bajpai et al., 2020), and markedly reduces the risk of pCIA (Martin et al., 2018; Kang et al., 2024). Yet, SC is less efficacious for many patients and exhibits reduced efficacy for certain highly genotoxic chemotherapy regimens (anthracyclines in particular).

We hypothesised previously that lack of clinical efficacy may be due to the severity of drug dose; however, cooling consistently rescues keratinocytes from chemotherapy drug concentrations equivalent to those reported in plasma during infusion (Al-Tameemi et al., 2014). Instead, a more plausible scenario is that low SC clinical efficacy results from inadequate cooling of the scalp in certain patients, particularly those treated with anthracycline regimens. In direct support of this are clinical observations that the subcutaneous scalp temperature reached in patients is a key determinant of efficacy (Komen et al., 2013). Importantly, scalp skin temperature during SC varies widely from 10°C to 31°C, and successful cooling (defined by use of no head-cover/wig) is associated with patients whose scalp has reached an “optimal” mean skin temperature of 18°C (Komen et al., 2016).

We have previously provided experimental evidence that cooling rescues human keratinocytes from taxane and anthracycline chemotherapy drug-mediated cytotoxicity in a temperature-dependent manner and that one of the mechanisms of cytoprotection involves inhibition of drug uptake. Such studies involved primary (NHEKs) and immortalised (HaCaTa) keratinocytes, as well as HF-derived matrix keratinocytes and ORSK cells (Al-Tameemi et al., 2014; Dunnill et al., 2020). Using such *in vitro* 2D-cell monoculture models, we have now extended our observations by demonstrating that: a) optimal cooling suppresses cellular ROS generation triggered by chemotherapy drugs, b) use of an antioxidant potentiates the cytoprotective effect of cooling, whilst c) combined use of ROS-scavenging antioxidants with sub-optimal cooling (26°C) confers protection that is equivalent to that observed under optimal (18°C) conditions. Thus, this combinatorial approach functionally compensates for “inadequate” cooling and restores cytoprotection efficacy.

However, because our current and previous such studies arguably employed relatively reductive-in-nature *in vitro* cell models, here we have utilised *ex vivo* human HF cultures as a more physiologically relevant model (Paus et al., 2013). We now show, for the first time, that cooling prevents drug-mediated cytotoxicity and rescues human HFs from drug-mediated cellular damage. Optimal cooling conditions suppressed HF entry into catagen and prevented loss of matrix keratinocyte proliferation and blocked apoptosis in a temperature-dependent manner. Cooling significantly derepressed hair shaft elongation, whilst also abrogating pigmentary abnormalities associated with chemotherapy-mediated HF toxicity.

Biologically interesting, too, was the observation that cooling alone in drug-untreated cultures (for 2-h in a 4-day culture period) consistently caused an increase in % Ki-67+ and decrease in % TUNEL+ cells in HF organ cultures assessed on Day 4. This is in concordance with our findings using NHEK and HaCaTa cells (here and previously (Al-Tameemi et al., 2014)), that a 2-h cooling period in control cultures caused detectable increases in viability when assessed 72-h post-treatment. This observation might appear counter-intuitive, as one would hypothesise that cooling would decelerate cell proliferation and/or metabolism (Dunnill et al., 2018). In fact, we have evidence that cooling temporarily triggers cell-cycle deceleration (increased G1 and decreased in G2/M phases) in both NHEK and HF matrix keratinocytes during the 2-h cooling period, yet cells rapidly resume cell-cycle progression after the cooling period (our unpublished observations). Instead, when proliferation is assessed in HFs and keratinocyte cultures 4-day or 72-h post-treatment, respectively, HF organ cultures and keratinocyte monocultures demonstrate improved viability in comparison to controls. It is tempting to speculate that these effects may be explained by attenuation of spontaneous “cell culture stress” related to basal ROS, a notion that is in line with our observation of reduction of ROS by cooling alone (Figure 1B). Irrespectively, cooling undoubtedly leaves its beneficial, biological “mark” on both keratinocytes and human HF organ cultures.

In agreement with our findings using *in vitro* cultured keratinocytes, use of antioxidant in conjunction with cooling provided a powerful combination that compensated for the lower efficacy of sub-optimal cooling in protecting *ex vivo* cultures of HFs. Importantly, the inability of antioxidants alone to rescue from drug toxicity (in keratinocytes and in HF cultures) strongly indicates that such compounds alone are highly unlikely to become a substitute for cooling. Instead, their use appears transformative in providing full protection at sub-optimal cooling for highly cytotoxic drugs. We believe that future use of antioxidants (in the form of a topical product applied to scalp skin) in conjunction with cooling represents an exciting treatment opportunity to compensate for clinical scenarios where some patients may not adequately respond to cooling (i.e., scalp temperature does not drop to optimal levels). This may also be highly beneficial for patients treated with more than one chemotherapy regimen, as such combinations are more potent and cooling exhibits lower protection efficacy both *in vitro* (Al-Tameemi et al., 2014) and in the clinic (van den Hurk et al., 2012). Moreover, as the antioxidants investigated demonstrated differential efficacies against 4HC and doxorubicin, our findings may also pave the way for the design of several cytoprotective combinations and raise the exciting prospect of chemotherapy regimen-specific targeting.

A significant proportion of non-targeted chemotherapeutics exert their anticancer effects via direct or indirect (secondary) induction of oxidative stress/ROS-mediated cell injury, as these drugs “push” cancer cells past a ROS-dictated lethal pro-apoptotic threshold (Dunnill et al., 2017) to induce death. Similarly, it is the induction of oxidative stress that accounts for the toxicity of these agents to HF matrix keratinocytes. As Nrf2 appears to play an important role in protecting matrix keratinocytes from ROS-mediated toxicity and catagen induction (Haslam et al., 2017), it is possible that the antioxidants used in this study may have activated Nrf2 and led to cytoprotection. Although we have molecular evidence that some of these (such as RV) induce Nrf2 activation in NHEK cells (our unpublished observations), antioxidant alone could not adequately protect from drug toxicity; this was observed not only for NAC, but also for RV and TX (of note, the antioxidant concentrations used were maximal tolerated doses for keratinocytes). Moreover, we employed a panel of compounds that utilise different mechanisms to exert their ROS-scavenging abilities. NAC accelerates glutathione biosynthesis and acts as a direct scavenger of free oxygen radicals, MT is a highly-specific scavenger of mitochondrial superoxide ions, RV exhibits dual protection against oxidative stress (active superoxide scavenger and Nrf2 activator), whilst TX is a peroxyl/alkoxyl radical scavenger, potent lipid peroxidation inhibitor and activates the Nrf2 pathway (Trueb, 2009; Liu et al., 2011; Pazdro and Burgess, 2012; Minter et al., 2020; Alva et al., 2022). These notions are significant, as they imply that additional mechanisms may be involved in the ability of cooling to cytoprotect, or (and perhaps more plausibly) that Nrf2-mediated cytoprotection operates below the lethal ROS levels triggered by cytotoxic drugs; thus, the protective capacity of Nrf2 can only be “unmasked” in combination with cooling which reduces cytotoxic load by curtailing drug uptake (Dunnill et al., 2020). This hypothesis may explain why neither sub-optimal cooling nor antioxidant alone are adequate, yet only their combination prevents cytotoxicity.

We also note our essentially identical observations using keratinocyte monocultures and HF mini-organ cultures. Using both models, we have demonstrated here that: a) cooling prevents chemotherapy drug-mediated toxicity, b) the efficacy of cooling is temperature-dependent, c) combination of cooling with antioxidant enhances the cytoprotective effect of cooling, whilst d) addition of antioxidant under sub-optimal cooling conditions restores cytoprotection capacity. Collectively, our findings constitute strong supportive evidence for the efficacy of cooling alone to cytoprotect and they demonstrate the inability of sub-optimal cooling conditions to suppress cytotoxicity (thus potentially providing an explanation as to why scalp cooling in the clinic is not as effective for some patients). Equally importantly, our study has provided a novel, highly promising combinatorial approach that may effectively combat HF toxicity against severely cytotoxic chemotherapeutic agents. This approach has the potential to transform the clinical efficacy of scalp cooling in preventing CIA, including permanent-CIA (pCIA), and thus significantly enhance cancer patient short-term quality-of-life during their treatment as well as long-term cancer patient survivorship.

In conclusion, our study provides for the first time evidence that cooling protects both human keratinocytes and, more significantly, human hair follicles (HFs) from chemotherapy-induced damage by attenuating oxidative stress, and that this cytoprotective effect is markedly enhanced through the co-application of antioxidants. Importantly, we show that optimal cooling alone effectively suppresses intracellular ROS production and prevents key pathological features of chemotherapy-induced toxicity, including HF catagen entry, apoptosis, and pigmentary disruption. Notably, as part of our novel observations, we show that co-treatment with antioxidants significantly potentiates the cytoprotective capacity of sub-optimal cooling, suggesting that this approach may be harnessed to overcome insufficient scalp cooling efficacy in a clinical setting. Our systematic evaluation of multiple antioxidants has revealed several promising candidates in addition to NAC (Trolox, Resveratrol and MitoTEMPO) which exhibit potent synergy with cooling. Taken together, these findings support a novel, biological mechanism-based strategy for improving CIA prevention, especially in patients receiving highly cytotoxic regimens or achieving sub-optimal scalp temperatures during scalp cooling. Future translational work should focus on developing antioxidant formulations for topical application, as well as tailoring cytoprotective combinations to specific chemotherapeutic regimens to maximize HF cytoprotection, reduce or prevent CIA and transform patient quality-of-life.

## Data Availability

The original contributions presented in the study are included in the article/Supplementary Material, further inquiries can be directed to the corresponding author.

## Supplementary material

SUPPLEMENTARY FIGURE S1 Blockade of chemotherapy drug-induced ROS generation by cooling alone or in combination with the antioxidant NAC in HaCaTa cells. Cells were treated with **(A)** 30 µM 4-hydroxycyclophosphamide (4HC) or **(B)** 1 µM doxorubicin (Doxo) for 2-h alongside untreated cells (Control) at normal (37°C) and at cooling (26°C, 22°C and 18°C) conditions, and ROS generation was detected using H2DCFDA. Experiments were also performed for these conditions in the presence or absence of 400 μM N-Acetyl-L-cysteine (NAC). ROS detection after treatment with 4HC **(A)** and Doxo **(B)** was performed spectrophotometrically and expressed as relative fluorescence units (RFUs). Bars show mean RFU (±SEM) for three representative experiments each consisting of four to five technical replicates. Statistical significance (cooling temperature versus 37°C for each treatment) is denoted as *p < 0.05, **p < 0.01 and ***p < 0.001, whilst NS indicates non-significance (p > 0.05).

SUPPLEMENTARY FIGURE S2 Representative images from hair cycle stage analysis (presented in Figure 4). Human HF organ cultures were treated with 30 µM 4-hydroxycyclophosphamide (4HC) in the presence or absence of 5 mM N-Acetyl-L-cysteine (NAC) alongside untreated cultures (Control) at normal temperature (37°C), at sub-optimal (26°C) and at optimal (18°C) cooling conditions (see Figure 4 caption for full details). HFs were assessed for hair cycle stage on Day 4 post-treatment and images from representative HFs for the different treatments are provided. Treatment with 4HC caused catagen induction in HFs characterised by dermal papilla condensation (small and ball-like-shape), a “brush-like” shaped hair shaft and a pointed-end in the hair bulb. By contrast, untreated follicles and those protected by cooling alone or cooling in combination with antioxidant NAC remained in anagen, and such HFs were characterised by a normal dermal papilla and a wide-end hair bulb.

SUPPLEMENTARY FIGURE S3 Antioxidants with different mechanisms of action potentiate the ability of sub-optimal cooling to protect against drug-induced cytotoxicity in HaCaTa cells. Cells were treated with 1 µM doxorubicin (Doxo) for 2-h in the presence or absence of **(A)** 10 µM Propyl Gallate (PG), **(B)** 100 µM MitoTEMPO (MT), **(C)** 10 µM Resveratrol (RV) or **(D)** 100 µM Trolox (TX) alongside untreated cells (Control), as well as cells treated with antioxidant only, at normal (37°C) and sub-optimal cooling (26°C) conditions and cell viability was assessed 72-h post-treatment. Bars represent mean absorbance units (±SEM) for three independent experiments, with each consisting of four to five technical replicates. Statistical significance ([drug] vs. [drug + antioxidant] at different temperature conditions) is denoted as *p < 0.05, **p < 0.01 and ***p < 0.001.
