# Supplementary figures and images for: Prevention of chemotherapy drug-mediated human hair follicle damage: combined use of cooling with antioxidant suppresses oxidative stress and prevents matrix keratinocyte cytotoxicity
Source: Front Pharmacol. 2025 Jul 8;16:1558593. doi: 10.3389/fphar.2025.1558593 (PMC12279381; doi:10.3389/fphar.2025.1558593)

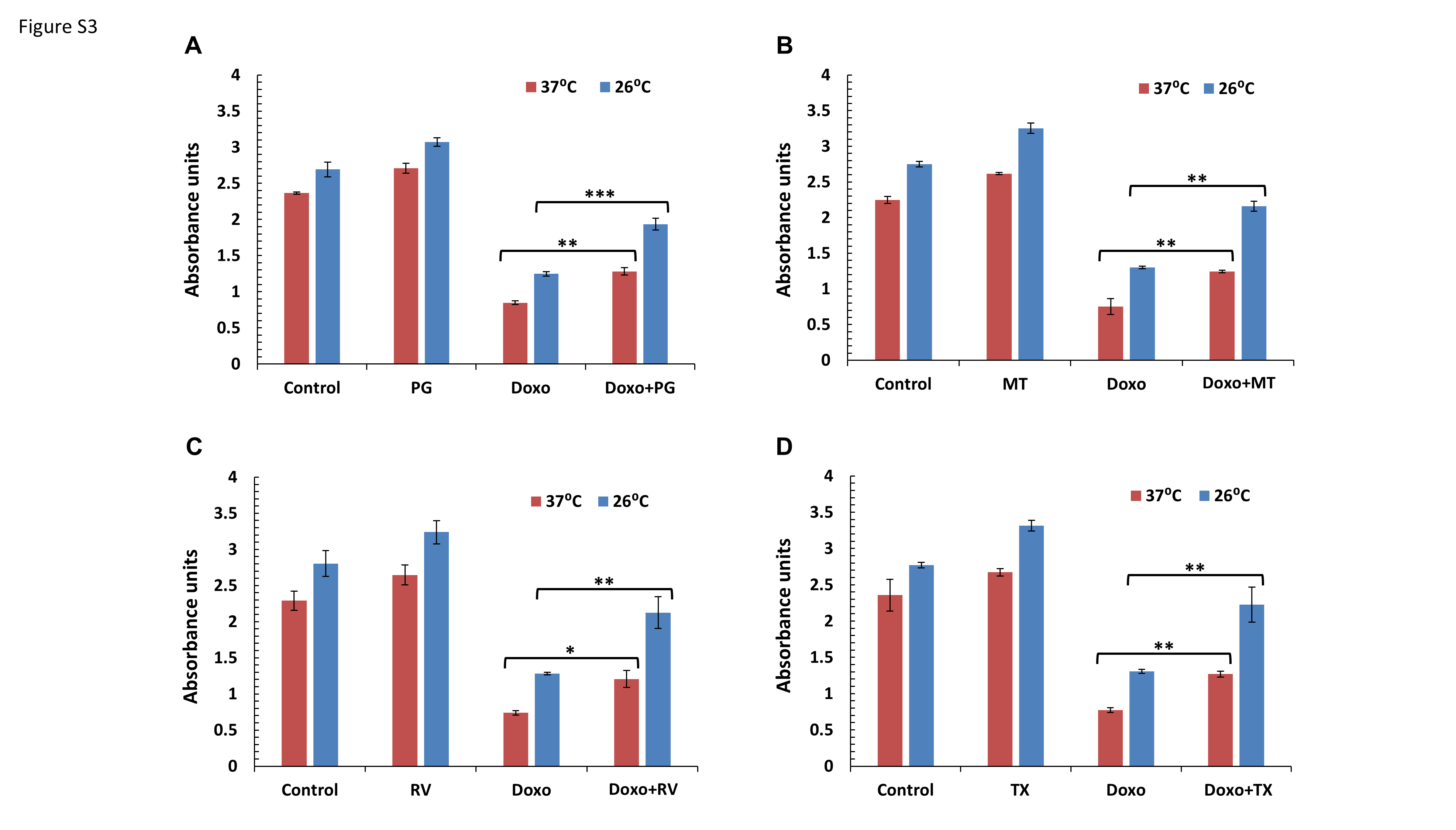

Supplement: Supplementary file 1 [file Image3.tif]

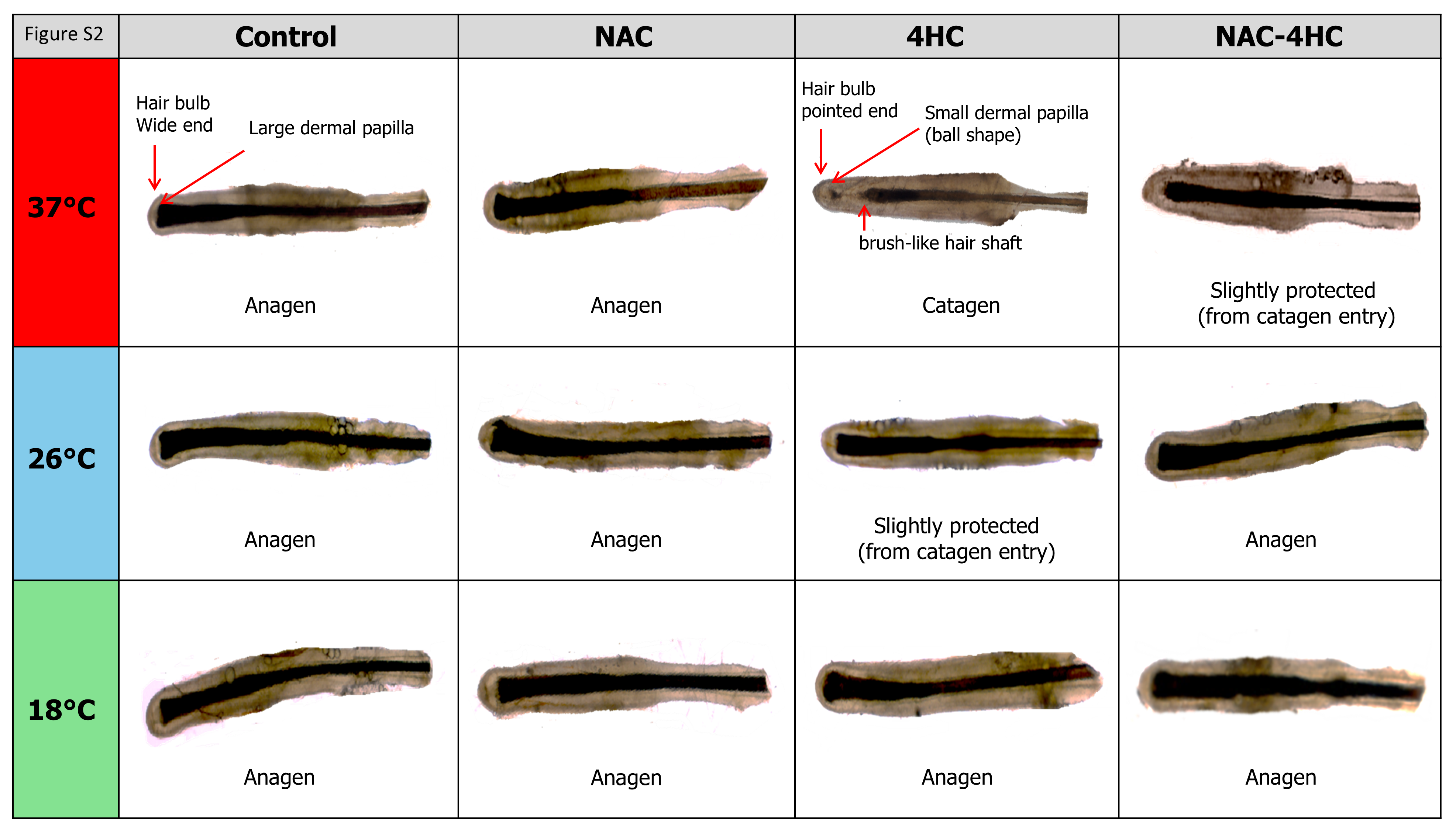

Supplement: Supplementary file 2 [file Image2.tif]

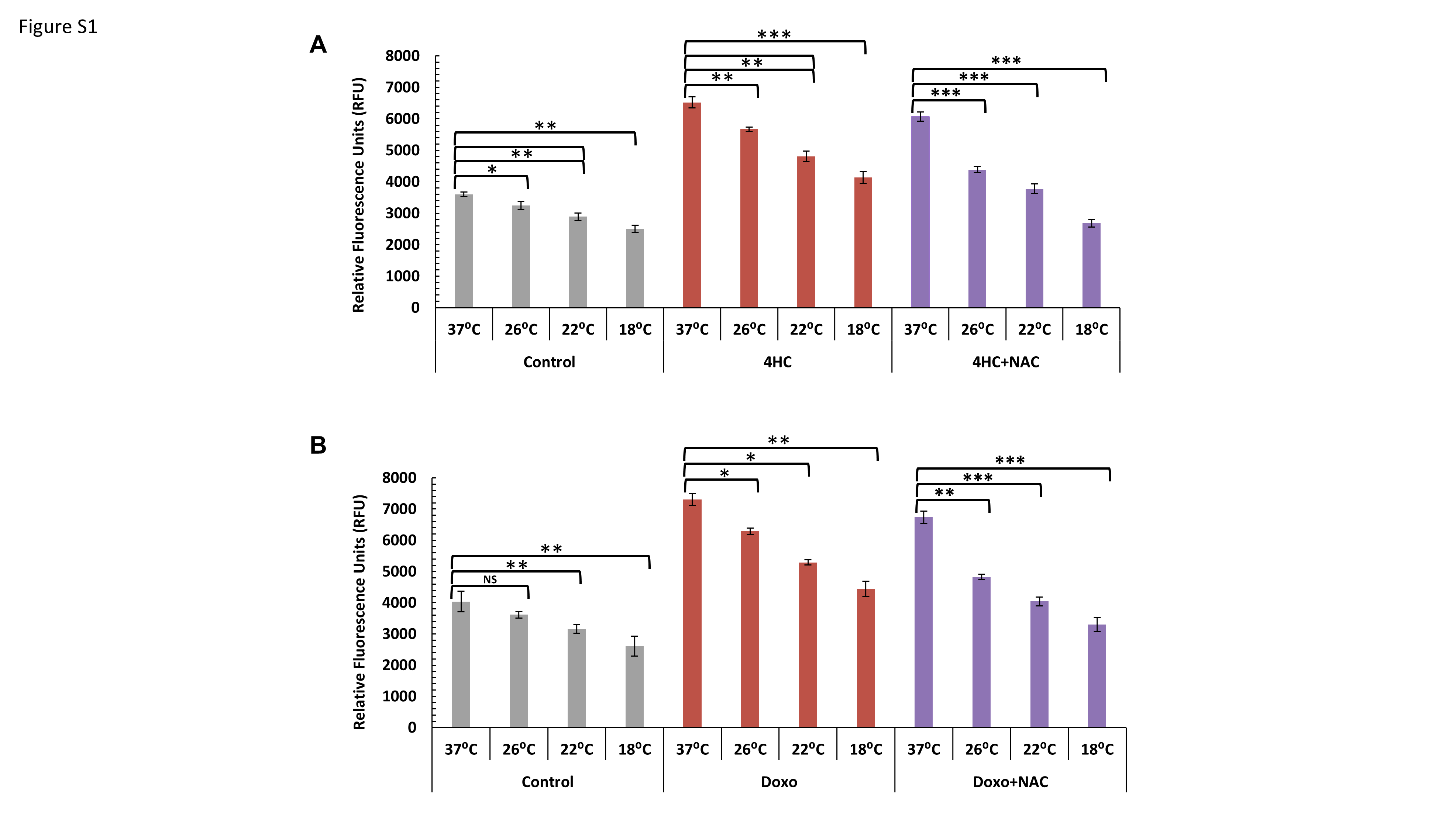

Supplement: Supplementary file 3 [file Image1.tif]
